# STEAP3 Affects Ovarian Cancer Progression by Regulating Ferroptosis through the p53/SLC7A11 Pathway

**DOI:** 10.1155/2024/4048527

**Published:** 2024-02-26

**Authors:** Yi Han, Lei Fu, Yan Kong, Changqing Jiang, Liying Huang, Hualing Zhang

**Affiliations:** Department of Gynecology, The Affiliated Hospital of Qingdao University, Qingdao 266000, Shandong, China

## Abstract

Ovarian cancer (OC) is a common malignant cancer in women with a low overall survival rate, and ferroptosis may be a potential new strategy for treatment. Six-transmembrane epithelial antigen of prostate 3 (STEAP3) is a gene closely related to ferroptosis, yet the role of STEAP3 in OC has not yet been thoroughly investigated. Using biological information analysis, we first found that STEAP3 was highly expressed in OC, which was significantly associated with poor prognosis of patients and was an independent prognostic factor. Through cloning, scratch, and transwell experiments, we subsequently found that knockdown of STEAP3 significantly reduced the proliferation and migration ability of OC cells. Furthermore, we found that knockdown of STEAP3 induced ferroptosis in OC cells by detecting ferroptosis indicators. Mechanistically, we also found that knockdown of STEAP3 induced ferroptosis through the p53/SLC7A11 signaling pathway. Through tumorigenic experiments in nude mice, we finally verified that the knockdown of STEAP3 could inhibit tumor growth in vivo by promoting ferroptosis through the p53 pathway. Overall, our study identified a novel therapeutic target for ferroptosis in OC and explored its specific mechanism of action.

## 1. Introduction

Ovarian cancer (OC) is one of the deadliest gynecologic malignancies, with approximately 239,000 new cases worldwide each year [1]. Due to the lack of specific biomarkers and typical clinical symptoms at the time of occurrence, early diagnosis of this disease has become difficult. As a result, over 60% of patients are already in the advanced stage when initially diagnosed with OC [2]. The main treatment options for most OC patients are surgery and systemic chemotherapy. However, the overall efficacy is poor and there is significant individual variation [3]. Although treatment options including immunotherapy have brought hope to advanced-stage patients in recent years, the 5-year survival rate for OC patients is only about 45% [4, 5]. Therefore, it is urgent and specific to explore more early detection and potential therapeutic targets for OC.

Ferroptosis, as a new form of cell death, has brought a new perspective to cancer treatment since its discovery in 2012 [6]. Therapies that have been targeting ferroptosis have been effective in many tumors, such as breast, prostate, and colorectal cancers [7–9]. In OC, Tang et al. [10] found that combination therapy with arsenic trioxide and olaparib increased lipid peroxidation and ultimately triggered iron death in OC cells, providing new mechanistic insights into drug therapy in platinum-resistant OC. Another recent study established CEBPG as a novel ferroptosis transcriptional regulator that regulates solute carrier family 7 member 11 (SLC7A11) expression in OC, with potential value in predicting patient prognosis and selection of therapeutic agents [11]. With increasing research on ferroptosis in OC, ferroptosis as a therapeutic strategy may help mitigate the progression of OC and address tumor resistance.

The six-transmembrane epithelial antigen of prostate 3 (STEAP3) encodes a multipass membrane metalloreductase, which can participate in Fe^3+^ transport as an iron transport protein [12]. Considering the close relationship between STEAP3 and cellular iron homeostasis, its role in a variety of cancers has been extensively studied [13]. A number of studies have found that upregulation of STEAP3 is strongly associated with poor prognosis in triple-negative breast cancer as well as gliomas, suggesting that STEAP3 may be used as a specific predictor [13, 14]. It has been also shown that STEAP3 expression is upregulated in renal cells and that it can affect the progression of renal cell carcinoma by regulating ferroptosis [15]. In addition, a novel ferroptosis-associated gene model containing STEAP3 in OC was able to predict the prognosis of OC patients accurately [16]. However, less has been reported about the relationship between STEAP3 and OC, and the specific mechanism of action of STEAP3 in OC is unknown.

In this study, we found that STEAP3 was abnormally expressed in various types of cancers by using information from several public databases. For OC, we performed further analysis and found that the expression level of STEAP3 in tumor tissues was significantly higher than that in normal tissues. Combined with clinical data, we also found that its high expression was significantly associated with poor prognosis. Follow-up experiments showed that the knockdown of STEAP3 could regulate ferroptosis to reduce the cell viability, proliferation, and migration of OC cells. In addition, we found a significant correlation between STEAP3 and p53 through correlation analysis and enrichment analysis. Further recovery experiments showed that the knockdown of STEAP3 could regulate the p53 signaling pathway to promote ferroptosis. In summary, our study demonstrated that STEAP3 is able to regulate ferroptosis in OC through the p53 pathway, which has the potential to be a novel approach for the treatment of OC.

## 2. Materials and Methods

### 2.1. Bioinformatics Analysis

Primary RNA sequencing (RNA-seq) data and corresponding clinical information were obtained from The Cancer Genome Atlas (TCGA, https://portal.gdc.cancer.gov/) and Genotype-Tissue Expression (GTEx, http://gtexportal.org/) databases, containing a total of 426 OC samples and 88 normal tissue samples. The expression matrices of the two independent datasets were batch normalized using the “sva” package of the R language. Independence of risk scores was determined using univariate and multivariate Cox analysis combined with clinical parameters (STEAP3, age, and grade).

### 2.2. Cell Culture and Transfection

Human OC cell lines SKOV3, A2780, and human normal ovarian surface epithelial (HOSE) cells were provided by the Typical Cultures Depository Center, USA. All cells were cultured in RPMI-1640 medium containing 10% FBS and 1% penicillin/streptomycin at an incubator temperature of 37°C and a CO_2_ concentration of 5%. Small interfering RNA (siRNA) was purchased from Sangon Biotech, and RNAiMAX transfection reagent was purchased from Thermo Fisher Scientific. Cells were inoculated in Petri dishes at a suitable density 1 day prior to transfection and then transfected according to the instructions of the reagent vendor.

### 2.3. In Vivo Xenograft Model

Sixteen male BALB/c nude mice (18–22 g) were purchased from Weitong Lihua Biotechnology (Beijing, China). Untreated SKOV3 cells and SKOV3 cells knocked down for STEAP3 were collected, resuspended using PBS (1 × 10^7^/ml), and then injected subcutaneously into the back of BALB/c nude mice. The nude mice were randomly divided into two groups, and the mice were executed after 30 days, then the tumors were excised and photographed. All animal experiments were performed in accordance with the NIH Guide for the Care and Use of Laboratory Animals and approved by the Laboratory Animal Welfare and Ethics Committee of the Affiliated Hospital of Qingdao University.

### 2.4. Reactive Oxygen Species (ROS) Levels

Reactive oxygen species (ROS) levels were detected using the ROS assay kit (Beyotime Biotechnology, S0033S, China). After cell treatment was completed, DCFH-DA was diluted to 10 *μ*M. Cells were incubated with DCFH-DA in a cell culture incubator at 37°C for 20 min. Cells were washed with PBS three times and collected. ROS levels were detected using the FITC channel of flow cytometry.

### 2.5. Superoxide Dismutase (SOD) Activity

The superoxide dismutase (SOD) activity of the cells was determined using the Total SOD assay kit (Beyotime Biotechnology, S0101S, China). Briefly, the cell and tissue samples were lysed and centrifuged to collect the supernatant, which was mixed with the working solution and incubated in a 37°C incubator for 0.5 hr. After incubation, the absorbance at 450 nm was measured using an enzyme marker.

### 2.6. Malondialdehyde (MDA) Content

The malondialdehyde (MDA) concentration of OC cells was determined using the Lipid Peroxidation MDA assay kit (Beyotime Biotechnology, S0131S, China). Briefly, the cell and tissue samples were lysed and centrifuged to collect the supernatant, which was subsequently mixed with the working solution and boiled in a water bath at 100°C for 20 min. The samples were cooled down and centrifuged to collect the supernatant, and the absorbance at 532 nm was determined using an enzyme marker.

### 2.7. Glutathione Peroxidase (GPX)

Cellular glutathione peroxidase (GPX) content was assayed according to the instructions of the Cellular GPX assay kit (Beyotime Biotechnology, S0056, China). Absorbance at 340 nm was measured using an enzyme meter.

### 2.8. Cell Viability

Cells were seeded in 96-well plates at a suitable density and incubated in an incubator for 0, 12, 24, and 48 hr. Cell viability was detected using the Cell Counting Kit-8 (CCK-8; Beyotime Biotechnology, C0037, China). Ten microliters of CCK-8 solution were added to each well and incubated at 37°C for 2 hr, and the absorbance was measured at 450 nm using an enzyme marker.

### 2.9. Quantitative Real-Time PCR

Total RNA was extracted using TRIzol (Thermo Fisher Scientific, USA), and was reverse transcribed into cDNA using PrimeScript™ RT Reagent Kit (TaKaRa, Japan). qRT-PCR analysis was performed using a CFX96 real-time PCR system. The GAPDH gene was conducted as an internal reference. The sequences of STEAP are listed below: forward, 5′-GTGAGCAACCCTACAGAGCA-3′; reverse, 5′-AAGAGGGAGGCCAGGTACTC-3′.

### 2.10. Western Blotting

The protein levels of STEAP3, p53, GPX4, SLC7A11, and acyl-CoA synthetase long-chain family member 4 (ACSL4) in cells were detected according to standard protocols, and GAPDH was used as an internal reference. After lysing the cells with RIPA lysate, protein concentrations were determined using the BCA protein assay kit (Beyotime Biotechnology, P0006, China). Samples were run on 7.5%–12.5% SDS polyacrylamide gel electrophoresis and then transferred to a PVDF membrane. The membranes were blocked with 5% skimmed milk for 1 hr at room temperature, followed by overnight incubation with primary antibody at 4°C. Antibody dilutions were STEAP3 (1 : 1,000, Abcam, UK), p53 (1 : 10,000, Abcam, UK), GPX4 (1 : 1,000, Abcam, UK), SLC7A11 (1 : 1,000, Abcam, UK), ACSL4 (1 : 10,000, Abcam, UK), and GAPDH (1 : 2,000, Abcam, UK). The next day, the proteins were incubated with secondary antibodies at room temperature for 1 hr. Proteins were developed with an ECL luminescent solution.

### 2.11. Wound Healing Assay

OC cells were seeded into 6-well plates at an appropriate density. When the cell density reached 70%–80%, the cell surface was scratched with a 100 ml pipette tip. Washed three times with PBS, the migration distance was recorded under a microscope after 24 hr.

### 2.12. Clonal Survival Assay

Cells were seeded into 6-well plates at a suitable density and cultured for 14 days. The cells were fixed using 4% paraformaldehyde for 20 min, then stained with 0.1% crystal violet for 30 min, and finally, the number of colonies was visually counted.

### 2.13. Statistical Analysis

Data were expressed as mean ± standard deviation (SD). Statistical analysis was performed using GraphPad Prism 8 software (GraphPad software, USA). Student's *t*-test and one-way analysis of variance (ANOVA) were used to determine the statistical significance of comparisons between groups. Differences of *P*  < 0.05 were considered statistically significant.

## 3. Results

### 3.1. STEAP3 Biological Information

**Figure 1:**
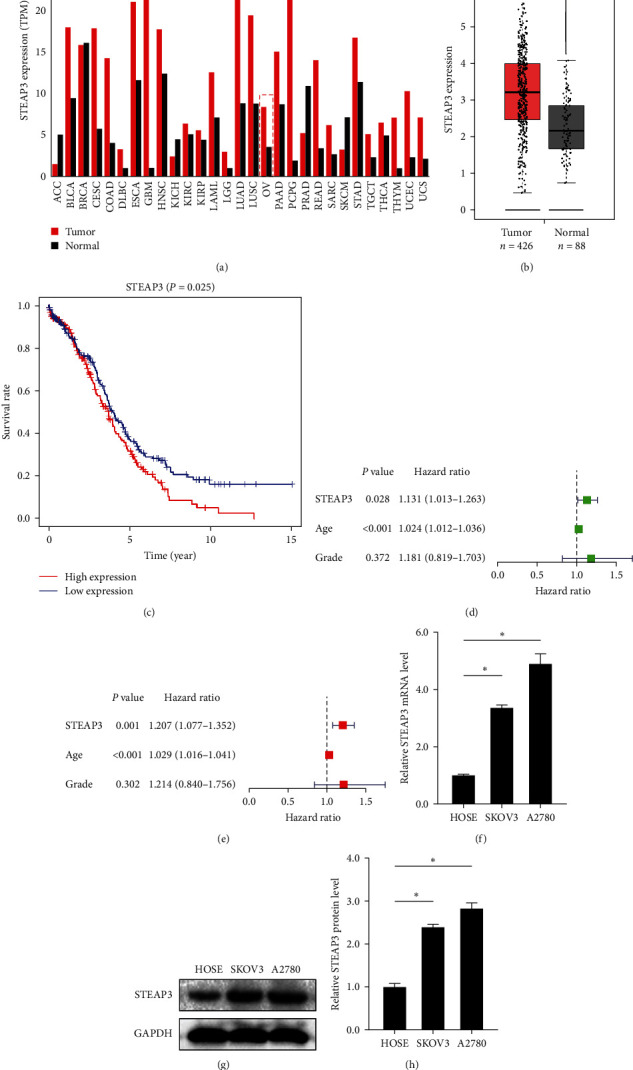
STEAP3 is highly expressed in tumors and associated with poor prognosis in ovarian cancer: (a) analysis from the GEPIA showing the expression of STEAP3 in common malignant tumors, (b) STEAP3 was generally highly expressed in ovarian cancer tissue samples, (c) patients were categorized into STEAP3 high-expression and low-expression groups according to the median. Kaplan–Meier's survival curve of patients with high or low STEAP3 expression level, (d, e) univariate and multivariate Cox regression analysis showed the correlation between clinicopathological factors and prognosis, (f) relative mRNA expression of STEAP3 in two OC cell lines (SKOV3 and A2780) and HOSE cell line was measured using qRT-PCR, and (g, h) representative blotting of STEAP3 in different OC cell lines, and quantification of STEAP3 protein levels relative to HOSE cell line. values are expressed as the mean ± SD, *n* = 3.  ^*∗*^*P* < 0.05.

By analyzing RNA-seq data from TCGA and GTEx databases, we found that STEAP3 was generally highly expressed in pan-cancer tissues (Figure 1(a)). In comparison to normal tissue samples, STEAP3 expression was significantly elevated in OC tissue samples (Figure 1(b)). Survival analysis showed that the survival rate was lower in the STEAP3-high expression group (Figure 1(c)). In addition, STEAP3 was shown to be an independent risk factor for OC along with age by Cox analysis (Figures 1(d) and 1(e)). Furthermore, the results of in vitro experiments showed that the expression level of STEAP3 in OC cell lines was significantly higher than in the HOSE cell line (Figure 1(f)–1(h)). What's more, we obtained tissue samples from OC patients from the clinic and detected the expression levels of STEAP3 in tumor tissues and paracancerous tissues. The results were consistent with previous findings (Figures S1(a) and S1(b)).

### 3.2. Knockdown of STEAP3 Inhibited Ovarian Cancer Cell Proliferation and Migration

**Figure 2:**
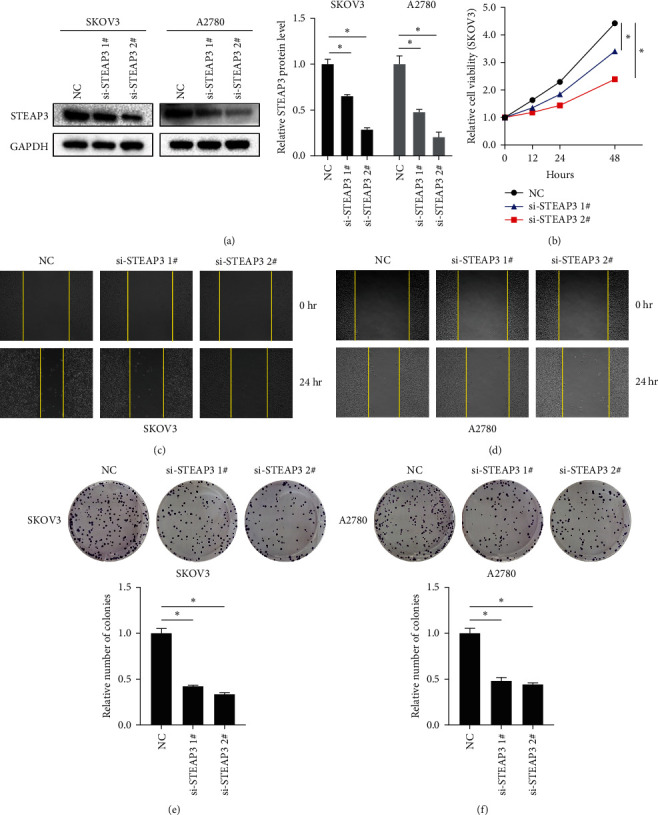
Knockdown of STEAP3 inhibits proliferation and migration of ovarian cancer cells: (a) protein expression levels of STEAP3 in SKOV3 and A2780 cells detected by western blot, (b) changes in SKOV3 cell viability detected by CCK-8, (c, d) representative images of wound healing in SKOV3 and A2780 cells, and (e, f) representative images of clonal survival of SKOV3 cells and A2780 cells and their quantitative analysis. Values are expressed as the mean ± SD, *n* = 3.  ^*∗*^*P* < 0.05.

To further explore the role of STEAP3 in OC, siRNA was used to transfect OC cell lines. The results showed that si-STEAP3 1# and si-STEAP3 2# successfully knocked down STEAP3 protein expression in SKOV3 cells and A2780 cells, with the knockdown efficiency of si-STEAP3 2# being even higher (Figure 2(a)). CCK-8 results showed that, in contrast to the NC group, si-STEAP3 significantly inhibited the expression of STEAP3 in SKOV3 cells and A2780 cell viability (Figure 2(b), Figure S2). Similarly, the knockdown of si-STEAP3 significantly reduced the invasive ability of SKOV3 cells and A2780 (Figures S3(a) and S3(b)). In addition, the knockdown of si-STEAP3 significantly slowed down wound healing in SKOV3 cells and A2780 cells (Figures 2(c) and 2(d)) and reduced cell clonal colonization (Figures 2(e) and 2(f)). Given that si-STEAP3 2# had a higher knockdown efficiency than si-STEAP3 1#, si-STEAP3 2# was used for all subsequent experiments.

### 3.3. Knockdown of STEAP3 Significantly Promoted Ferroptosis in Ovarian Cancer Cells

**Figure 3:**
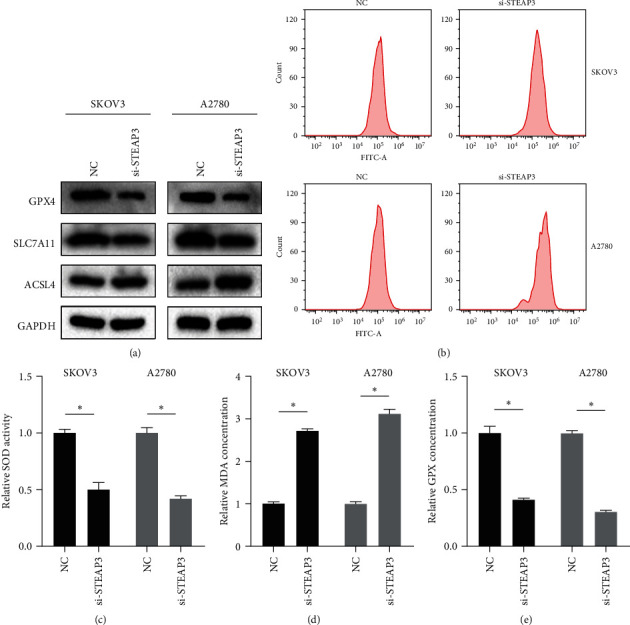
Knockdown of STEAP3 promotes ferroptosis in ovarian cancer cells: (a) the expression levels of ferroptosis-related proteins in SKOV3 cells and A2780 cells were detected by western blot, (b) the ROS levels in SKOV3 cells and A2780 cells were detected by flow cytometry, and (c–e) the SOD activity, MDA content, and GPX content in SKOV3 cells and A2780 cells were detected according to commercial kits. Values are expressed as the mean ± SD, *n* = 3.  ^*∗*^*P* < 0.05.

Subsequently, we further explored the possible mechanism of knocking down STEAP3 antitumor. We found that the knockdown of STEAP3 significantly increased the level of ferroptosis in SKOV3 cells and A2780 cells, and protein expression of GPX4 and SLC7A11 decreased, while ACSL4 expression increased (Figure 3(a)). Detecting ROS levels in OC cell lines by flow cytometry, we found that the knockdown of STEAP3 resulted in a significant increase in ROS levels in SKOV3 cells and A2780 cells (Figure 3(b)). In addition, the knockdown of STEAP3 led to a decrease in SOD, GPX activity, and an increase in MDA content (Figure 3(c)–3(e)).

### 3.4. Knockdown of STEAP3 Promoted Ferroptosis in Ovarian Cancer Cells through Activation of p53

**Figure 4:**
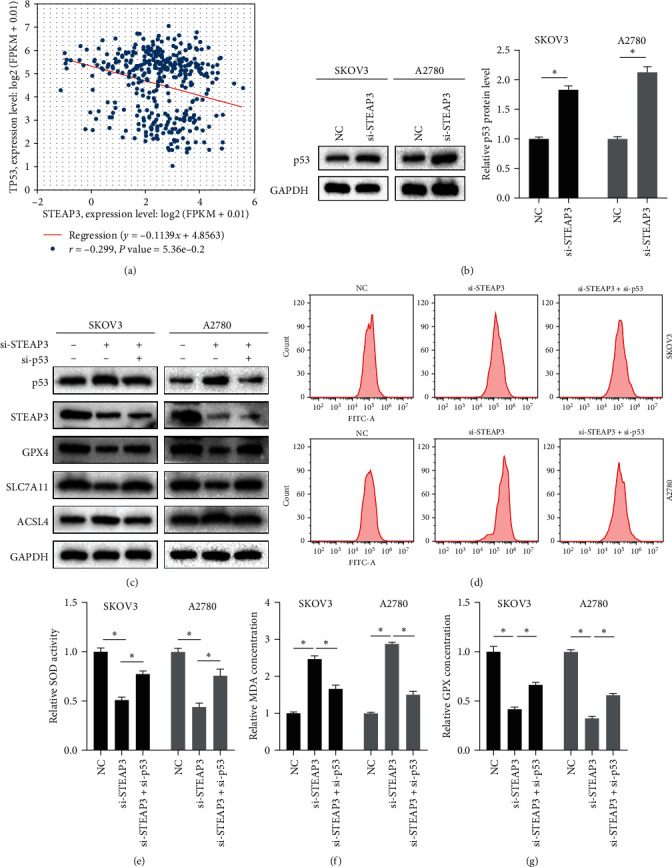
Knockdown of STEAP3 promotes ferroptosis in ovarian cancer cells through activation of p53: (a) a significant negative correlation between STEAP3 and p53 expression through bioinformatics analysis, (b) protein expression levels of p53 in SKOV3 and A2780 cells were detected by western blot, (c) expression levels of p53, STEAP3, and ferroptosis proteins in SKOV3 and A2780 cells were detected by western blot, (d) ROS levels, and (e–g) SOD activity, MDA content, and GPX content of SKOV3 cells and A2780 cells were detected according to commercial kits. Values are expressed as the mean ± SD, *n* = 3.  ^*∗*^*P* < 0.05.

To investigate how STEAP3 regulates ferroptosis in OC, we found that p53 was negatively correlated with the expression of STEAP3 through bioinformatics analysis (Figure 4(a)). Similarly, immunohistochemical results of clinical tumor tissues verified a significant negative correlation between STEAP3 and p53 expression (Figures S1(a) and S1(c)). Subsequently, we found that STEAP3 could be enriched in the p53 pathway by further GSEA enrichment analysis of the STEAP3 gene (Figure S4). In addition, p53 was recognized as a key factor regulating cellular ferroptosis, and we explored the association of STEAP3 with p53 in OC cells. Interestingly, we found that the knockdown of STEAP3 resulted in a significant increase in p53 protein expression (Figure 4(b)), while the knockdown of p53 had no effect on STEAP3 expression (Figure 4(c)). All of these results indicated that STEAP3 could regulate p53 expression and reduce the protein level of p53 in ovarian tumors. To investigate whether the role of knockdown of STEAP3 in promoting ferroptosis was dependent on p53, we found that knockdown of p53 significantly reduced ferroptosis in SKOV3 cells and A2780 cells, with a rise in protein expression of GPX4 and SLC7A11 and a decrease in ACSL4 expression (Figure 4(c)). In addition, SKOV3 cells and A2780 cells showed a decrease in ROS and MDA and a rise in SOD and GPX activities (Figure 4(d)–4(g)). Knockdown of p53 apparently partially eliminated the function of STEAP3 knockdown to promote ferroptosis in OC cells, thus knockdown of STEAP3 partially depended on the activation of p53 to play a role in promoting ferroptosis.

### 3.5. Inhibition of Ovarian Cancer Cell Proliferation by STEAP3 Knockdown was Reversed by Knockdown of p53

**Figure 5:**
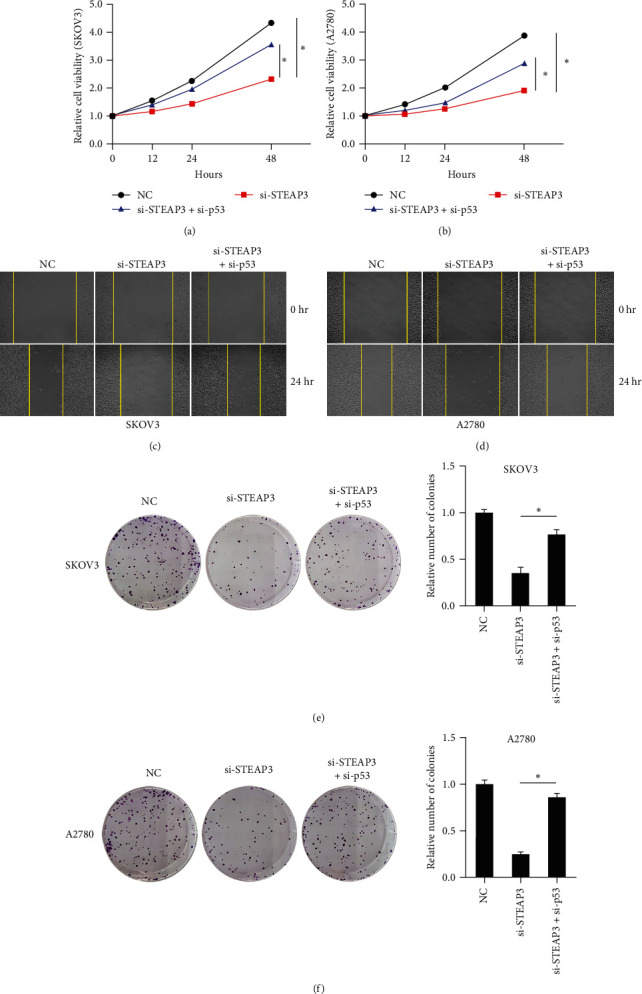
Knockdown of p53 promotes proliferation and migration of ovarian cancer cells: (a, b) changes in the viability of SKOV3 cells and A2780 cells detected by CCK-8, (c, d) representative images of wound healing of SKOV3 cells and A2780 cells, and (e, f) representative images of clonal survival of SKOV3 cells and A2780 cells and their quantitative analysis. Values are expressed as the mean ± SD, *n* = 3.  ^*∗*^*P* < 0.05.

We examined the effect of knocking down p53 on OC cell proliferation. The results showed that the knockdown of STEAP3 significantly reduced cell viability, which was reversed by the knockdown of p53 (Figures 5(a) and 5(b)). The knockdown of p53 reversed the ability of si-STEAP3 to inhibit the migration of SKOV3 cells and A2780 cells (Figures S5(a) and S5(b)). In addition, knockdown of p53 accelerated wound healing in SKOV3 cells and A2780 cells (Figures 5(c) and 5(d)), and in terms of cell clone formation, knockdown of p53 partially eliminated the function of knockdown of STEAP3 that could inhibit colony formation (Figures 5(e) and 5(f)). Thus, the inhibition of OC cell proliferation by knockdown of STEAP3 was dependent on p53 activation.

### 3.6. Knockdown of STEAP3 Inhibits Ovarian Cancer Cell Growth and Induces Ferroptosis In Vivo through p53 Pathway

**Figure 6:**
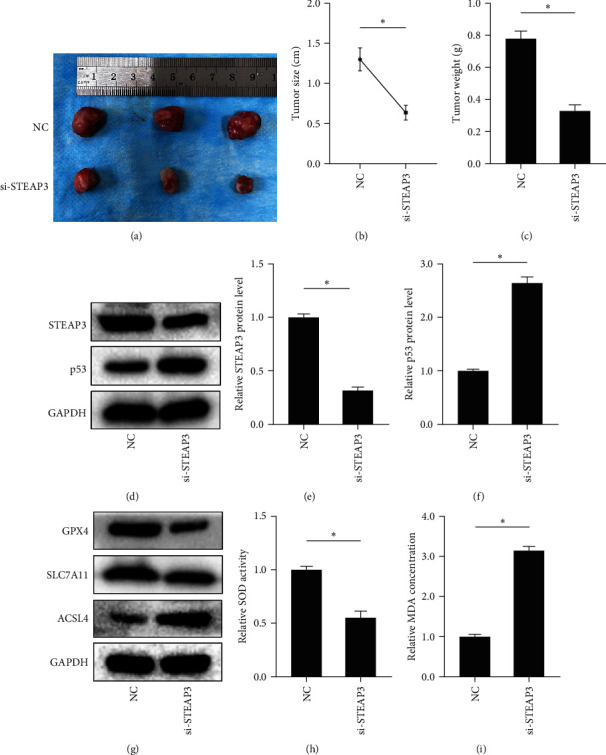
Knockdown of STEAP3 inhibits tumor growth and promotes ferroptosis: (a) xenograft tumors with different treatments, (b, c) size and weight of xenograft tumors in different treatment groups, (d–f) representative blotting and quantification of STEAP3 and p53 in different xenograft tumor, (g) the expression levels of ferroptosis-related proteins in different xenograft tumors were detected by western blot, and (h, i) the SOD activity and MDA content different xenograft tumors were detected according to commercial kits. Values are expressed as the mean ± SD, *n* = 3.  ^*∗*^*P* < 0.05.

To further confirm the effect of STEAP3 in OC cells in vivo, we established subcutaneous tumor models carrying normal SKOV3 cells and STEAP3 knockdown SKOV3 cells, respectively. We examined the effect of knocking down p53 on OC cell proliferation. Then, we measured the volume and weight of xenograft tumors. As shown in Figure 6(a)–6(c), STEAP3 knockdown significantly inhibited ovarian tumor growth. Similarly, STEAP3 knockdown was able to promote p53 expression (Figure 6(d)–6(f)). Moreover, protein expression of GPX4 and SLC7A11 decreased, while ACSL4 expression increased (Figure 6(g)). And the changes in SOD and MDA also suggested that knockdown of STEAP3 promoted ferroptosis (Figures 6(h) and 6(i)). The results showed that the knockdown of STEAP3 significantly inhibited OC cell growth by promoting ferroptosis through the p53 pathway in vivo.

## 4. Discussion

STEAP3 is a member of the six-transmembrane epithelial antigen of the prostate family which is a class of structurally similar cell surface membrane proteins with six transmembrane structural domains [17]. STEAP3 consists of a six-transmembrane structural domain of the COOH-terminal structural domain and a cytoplasmic N-terminal oxidoreductase structural domain, which are closely related to iron transport and reduction [18]. Studies have shown that STEAP3 is involved in cellular redox, inflammatory secretion, proliferation, and differentiation, closely related to a variety of physiological and pathological activities [19]. Aberrant expression of STEAP3 is an important factor in disease progression in many tumors. STEAP3 was able to promote glioma migration and invasion and predicted poor prognosis, suggesting that STEAP3 is a potential target for glioma diagnosis and treatment [20]. In another study of liver cancer, researchers found that STEAP3 promotes cancer cell proliferation by facilitating nuclear transport of EGFR [21]. It has also been found that STEAP3 is significantly upregulated in renal cell carcinoma and is associated with poor prognosis [22]. In addition, STEAP3 is involved in the remodeling of the extracellular matrix and the formation of tumor immune microenvironment in renal cancer to promote tumor metastasis and immune evasion [23]. Recent studies suggest that STEAP3 may be associated with poor prognosis in OC [24]. In our study, we downloaded information from public databases and found that STEAP3 expression was significantly higher in many tumors than in normal tissues, especially glioblastoma, pheochromocytoma, thymoma, and OC. Through further analysis, we identified that STEAP3 expression in OC was negatively correlated with survival time and was an independent predictor. Subsequent experimental validation showed that knockdown of STEAP3 significantly reduced the proliferation and migration of OC cells.

Ferroptosis, a novel iron-dependent mode of death mediated by the accumulation of ROS and lipid peroxidation products, opens a new door for the treatment of OC [25]. The curcumin derivative NL01 was revealed to reduce HCAR1/MCT1 expression in OC cells and consequently induced ferroptosis in tumor cells, suggesting that NL01 is promising for application in OC therapy. Other studies found that eriodictyol regulated ferroptosis in OC to exert an antitumor effect. Further experiments showed that eriodictyol induced ferroptosis and mitochondrial dysfunction through the Nrf2/HO-1/NQO1 pathway [26]. SLC7A11 and GPX4 are the most important regulators of ferroptosis. GPX4 reduces peroxidized lipids and protects cells from lipid peroxidation-induced ferroptosis [27]. SLC7A11 is an important component of the cystine/glutamate reverse transporter (Xc−) system. Its abnormality affects cysteine transport in cells, thus indirectly regulating GPX4 [28]. There is evidence that high coexpression of SLC7A11 and GPX4 serves as a predictor of platinum resistance and poor prognosis in OC patients [29]. Previous studies have shown that the knockdown of STEAP3 affects ferroptosis and renal cell carcinoma progression by regulating the Xc− system [15]. Similarly, our study found that the knockdown of STEAP3 decreased the expression of SLC7A11 and GPX4 in OC cells. In addition, knockdown of STEAP3 promoted the expression of the ferroptosis protein ACSL4. Further assays revealed that the knockdown of STEAP3 significantly increased ROS and MDA, as well as decreased the levels of GSH and SOD in OC cells. The above results confirmed that the knockdown of STEAP3 induced the development of ferroptosis in OC.

The p53 gene is activated in response to various stress signals (DNA damage, hyperproliferative signals, hypoxia, oxidative stress, and ribonucleotide depletion, etc.) and triggers cell cycle arrest and apoptosis. As a clear and important oncogene, inactivation of p53 occurs frequently during the development of a number of tumors [30]. Moreover, p53 is also an important regulator of ferroptosis [31]. Previous studies revealed that p53 inhibited the expression of SLC7A11, which in turn inhibited cystine uptake and induced ferroptosis [32]. In OC, MEX3A mediated degradation of p53 to inhibit ferroptosis and promote OC tumor progression [33]. In another study on the application of PARP inhibitors in OC, researchers found that PARP inhibition promoted ferroptosis by inhibiting SLC7A11 in a p53-dependent manner [34]. Passer et al. [35] discovered that STEAP3 and p53 expression were complementary and that STEAP3 could regulate p53 expression. In addition, STEAP3 knockdown induced ferroptosis in renal cell carcinoma through the p53/SLC7A11 pathway [15]. In the current study, a significant negative correlation between STEAP3 and p53 expression was observed in OC. Further exploration revealed that STEAP3 knockdown significantly increased P53 expression and p53 inhibition was able to partially reverse STEAP3 knockdown-induced ferroptosis in two OC cell lines. Similarly, p53 inhibition partially restored the altered proliferative and migratory capacities of OC cells caused by STEAP3 knockdown. Furthermore, tumorigenic experiments in nude mice showed that the knockdown of STEAP3 significantly inhibited the growth of OC cells by promoting ferroptosis through the p53 pathway.

In conclusion, our study systematically investigated the expression of STEAP3 in OC and its impact on prognosis. In addition, a series of experiments confirmed that STEAP3 can regulate OC progression by affecting ferroptosis through the p53/SLC7A11 pathway. Our study identified a novel mechanism for the regulation of ferroptosis in OC and provides new insights into the early diagnosis and treatment of OC.

## Data Availability

All data are available from the corresponding author.

## Supplementary Materials

Supplementary Materials Figure S1: expression levels of STEAP3 and p53 in ovarian cancer tissues. Figure S2: the viability of A2780 cells. Figure S3: transwell experiment to detect SKOV3 and A2780 cell migration ability. Figure S4: STEAP3 gene function enrichment analysis. Figure S5: transwell experiment to detect SKOV3 and A2780 cell migration ability.
