# Supplementary material for: STEAP3 Affects Ovarian Cancer Progression by Regulating Ferroptosis through the p53/SLC7A11 Pathway
Source: Mediators Inflamm. 2024 Feb 26;2024:4048527. doi: 10.1155/2024/4048527 (PMC10911874; doi:10.1155/2024/4048527)
Supplement: Supplementary Materials — Figure S1: expression levels of STEAP3 and p53 in ovarian cancer tissues. Figure S2: the viability of A2780 cells. Figure S3: transwell experiment to detect SKOV3 and A2780 cell migration ability. Figure S4: STEAP3 gene function enrichment analysis. Figure S5: transwell experiment to detect SKOV3 and A2780 cell migration ability. [file 4048527.f1.docx]

**Supplementary Materials**


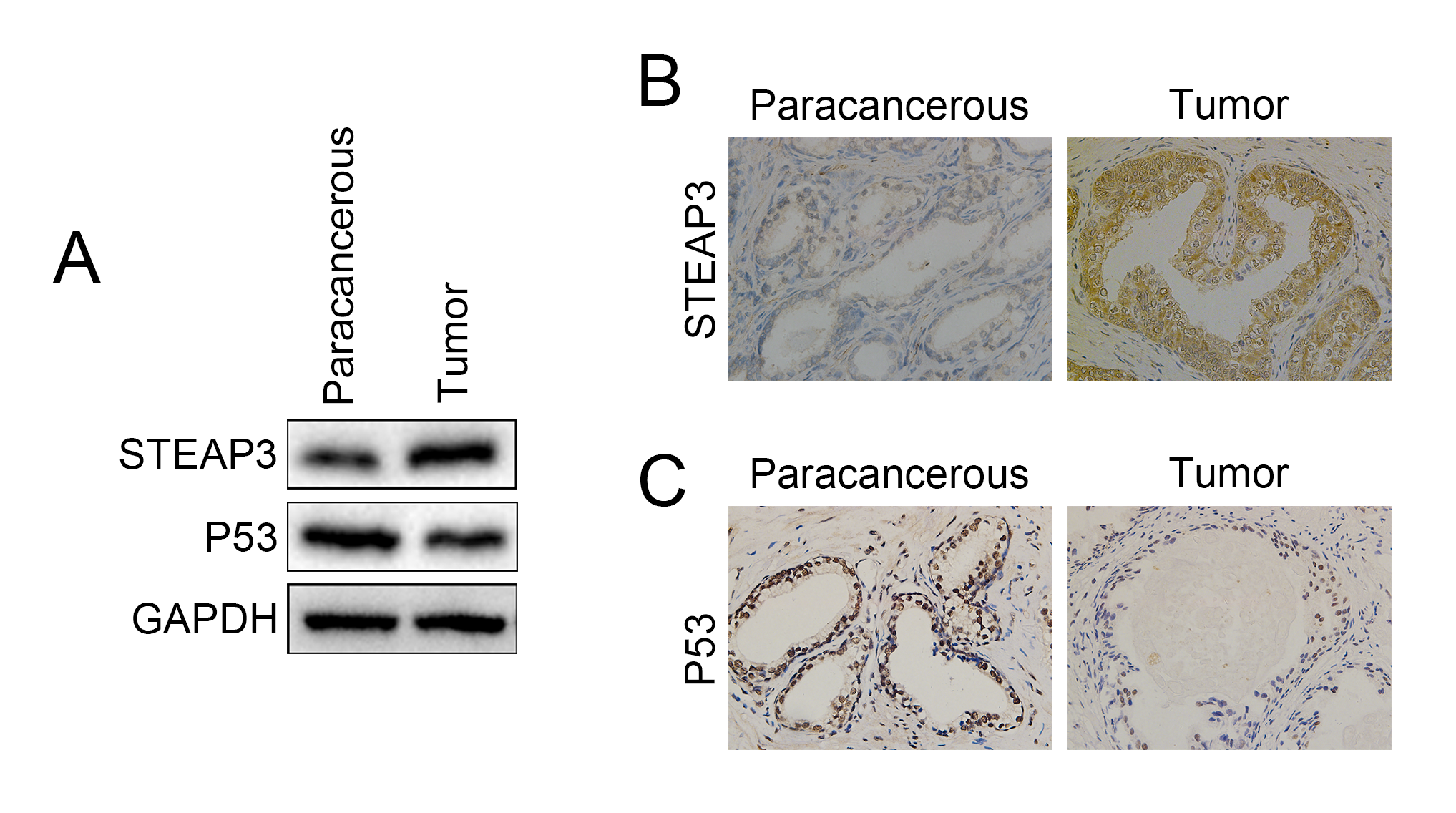


**Supplementary Figure 1 Expression levels of STEAP3 and P53 in ovarian cancer tissues.**

(A) The protein levels of STEAP3 and P53 in ovarian cancer tissues were detected by Western blot; (B, C) The protein levels of STEAP3 and P53 in ovarian cancer tissues were detected by immunohistochemistry.


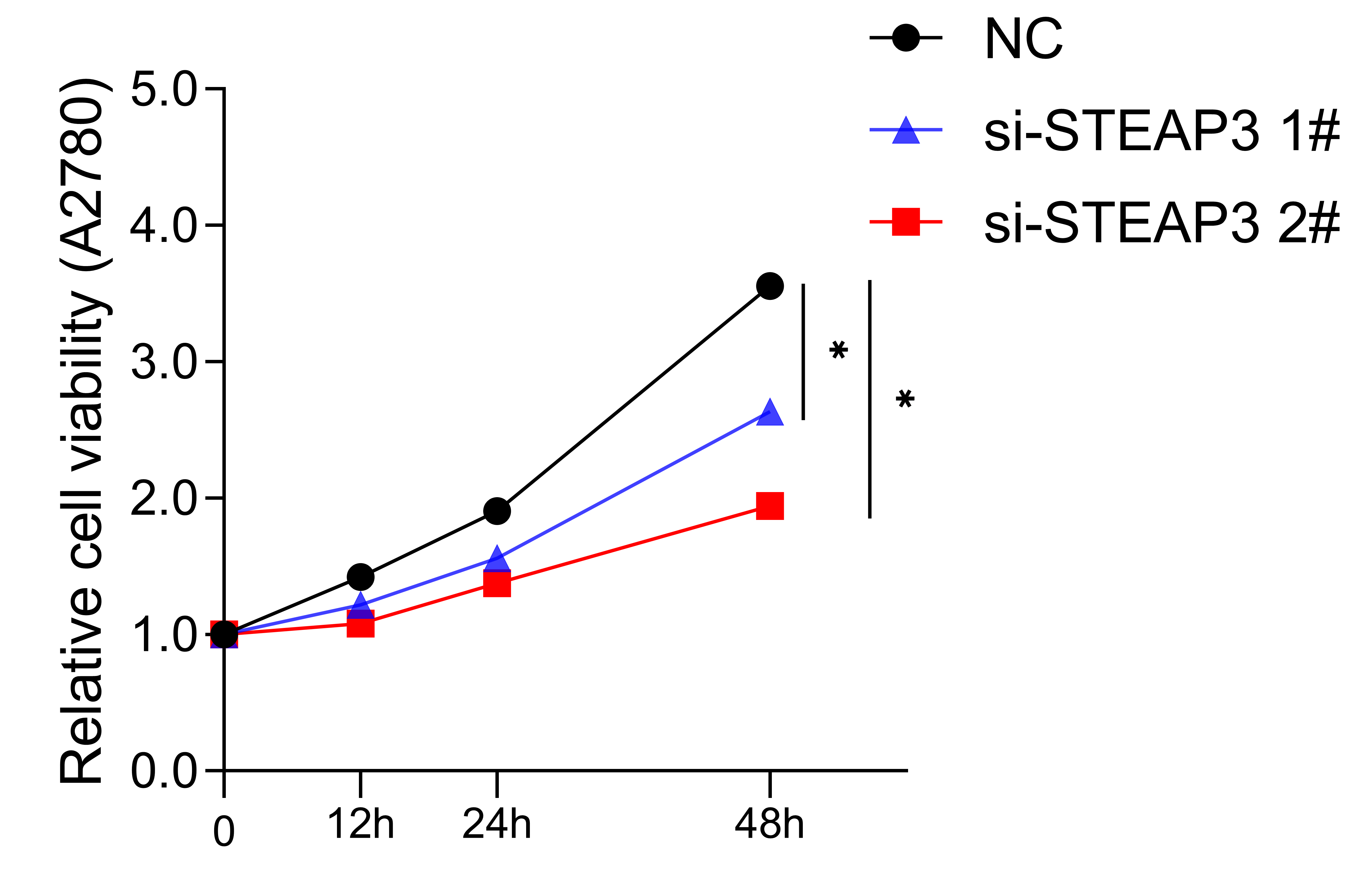


**Supplementary Figure 2 The viability of A2780 cells.**

Changes in A2780 cell viability were detected by CCK-8. Values are expressed as the mean ± SD, n = 3. ^∗^P < 0.05.


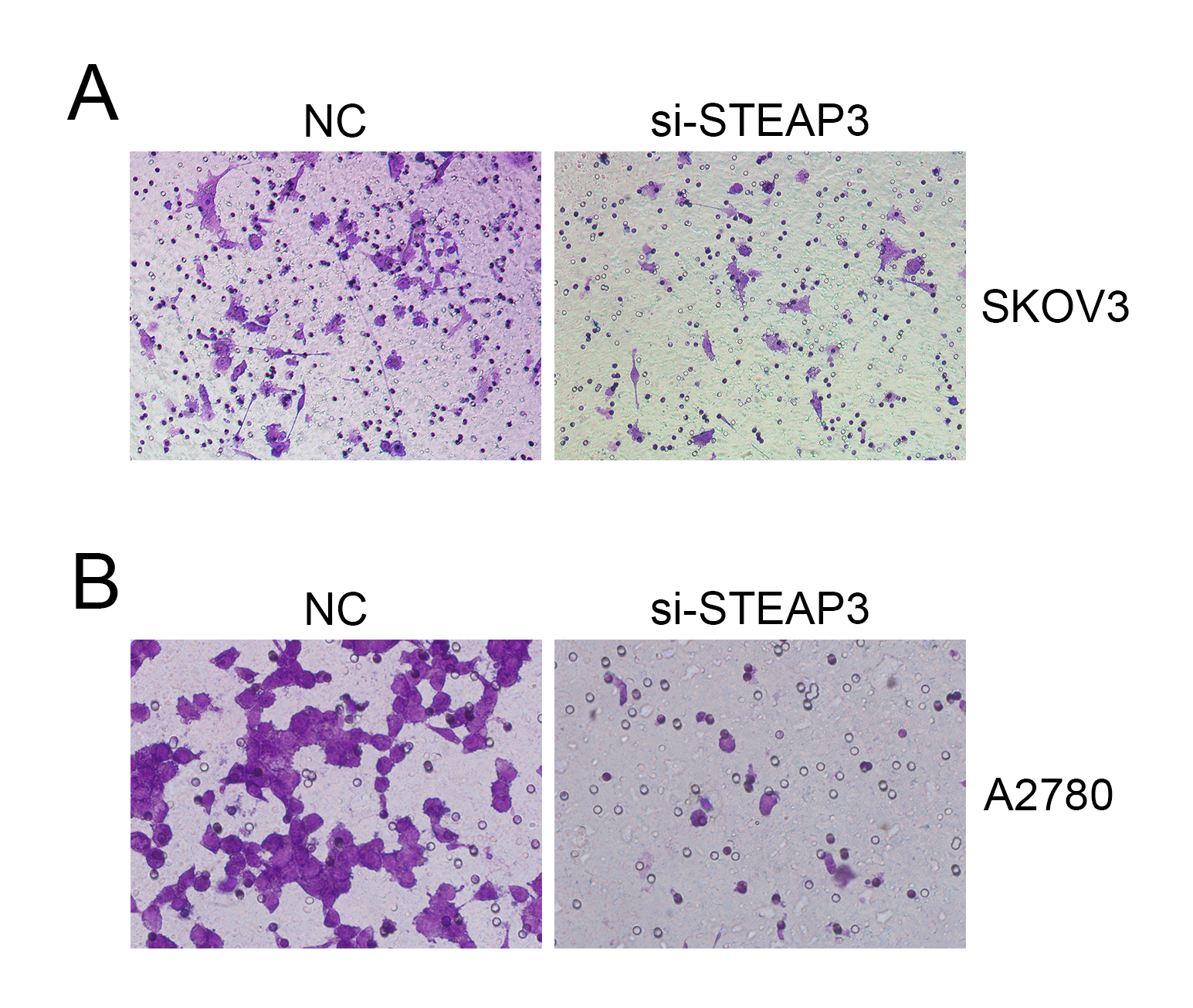


**Supplementary Figure 3 Transwell experiment to detect SKOV3 and A2780 cell migration ability.**

(A) SKOV3 cell migration ability; (B) A2780 cell migration ability. si-STEAP3 is si-STEAP3 2#.


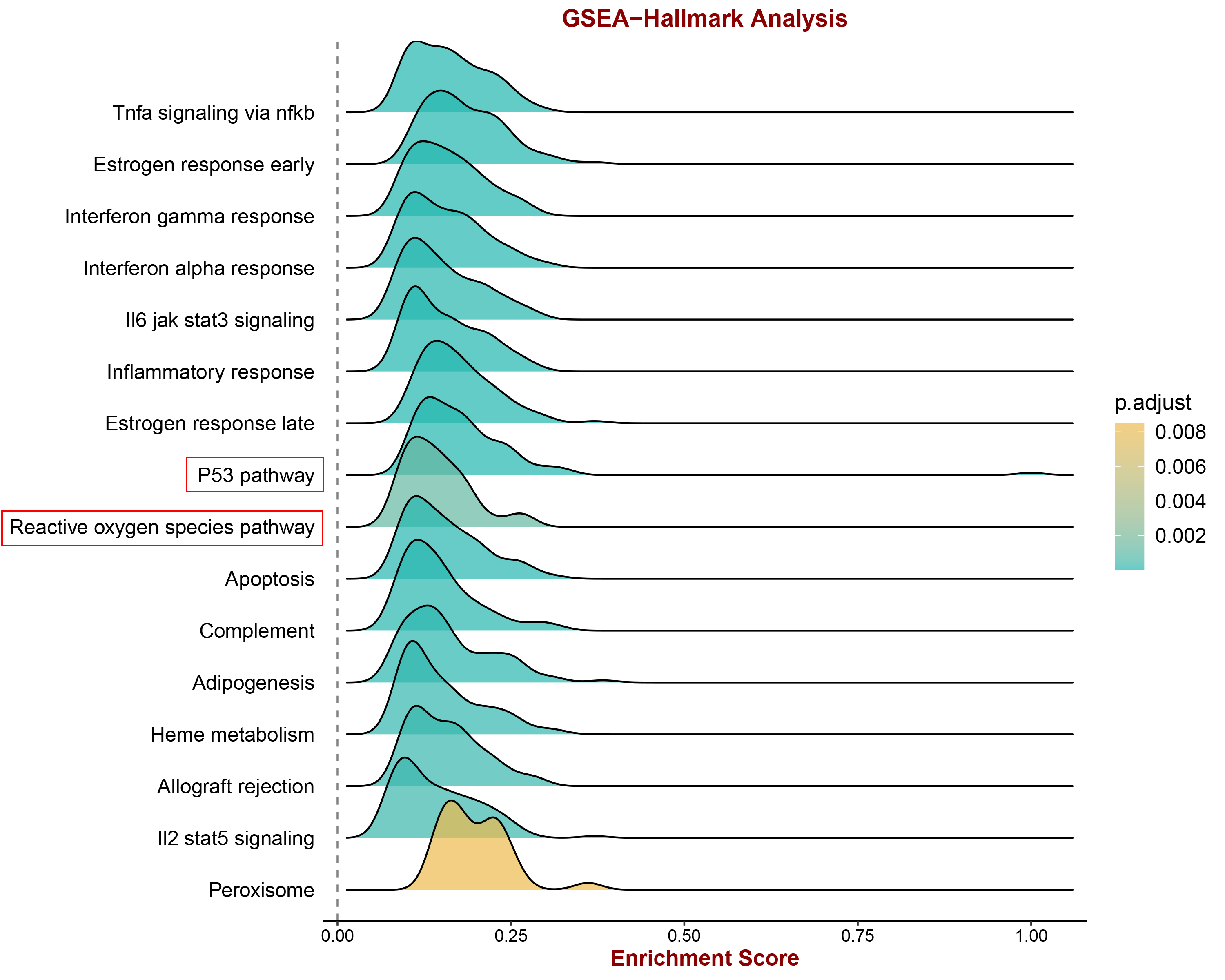


**Supplementary Figure 4 STEAP3 gene function enrichment analysis.**

STEAP3 was associated with the P53 pathway and the reactive oxygen species pathway by gene set enrichment analysis (GSEA).


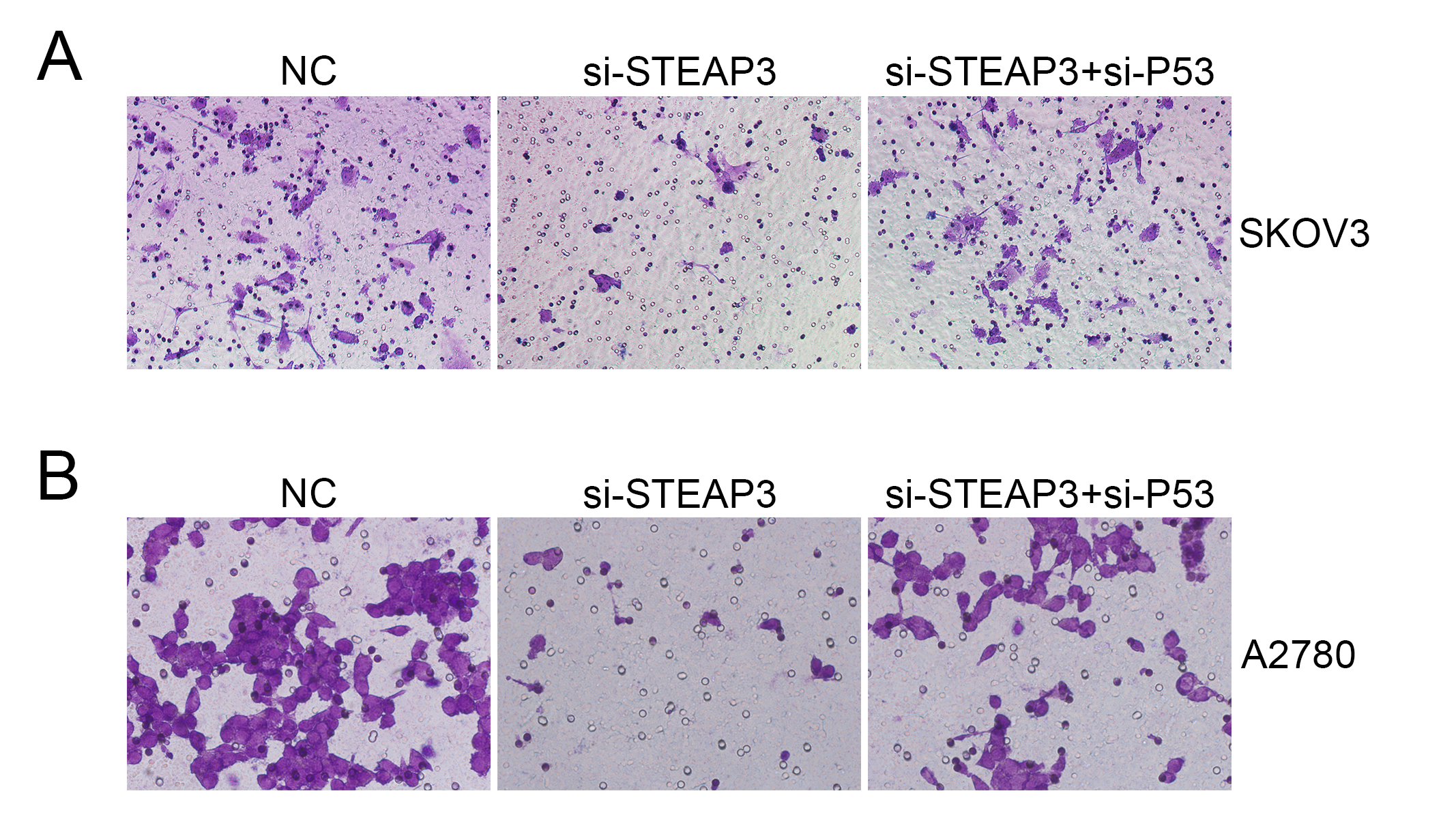


**Supplementary Figure 5 Transwell experiment to detect SKOV3 and A2780 cell migration ability.**

1. SKOV3 cell migration ability; (B) A2780 cell migration ability. si-STEAP3 is si-STEAP3 2#.

**Supplementary Materials and Methods**

**1. Immunohistochemistry**

Human ovarian cancer tissues and paracarcinoma tissues were paraffin-embedded, cut into 4-μm-thick sections, and stained with Immunohistochemistry UltraSensitive Kit (Maixin Biologicals, KIT-9710, China) according to the instructions from the reagent vendors.The dilution concentration of STEAP3 (PROTEINTECH NORTH AMERICA, USA) primary antibody was 1:50, and the dilution concentration of P53 (Abcam , UK) primary antibody was diluted at a concentration of 1:50.

**2. Transwell experiment**

SKOV3 and A2780 cells were inoculated in transwell chambers, which were removed after 24 h of culture, fixed with 4% paraformaldehyde (Beyotime Biotechnology, China) for 15 min, and washed with PBS for three times. Then the transwells were stained with 0.1% crystal violet (Beyotime Biotechnology, China) for 20 min at room temperature, washed three times with PBS and imaged under a microscope.
